# Isolated Central Nervous System Tuberculosis Presenting as Progressive Encephalopathy: A Report of a Biopsy-Proven Case

**DOI:** 10.7759/cureus.111645

**Published:** 2026-06-28

**Authors:** Linnet Leon Chaviano, Leen Daghir, Rawan Zeizzoun, Amer Zeizoun, Indulekha Gopal

**Affiliations:** 1 Department of Internal Medicine, Broward Health North, Pompano Beach, USA; 2 Faculty of Medicine, Ain Shams University, Cairo, EGY; 3 Department of Infectious Disease, Broward Health North, Pompano Beach, USA

**Keywords:** brain biopsy diagnosis, central nervous system tuberculosis, chronic encephalopathy, leptomeningeal enhancement, tuberculous meningitis

## Abstract

Central nervous system (CNS) tuberculosis (TB) is an uncommon but severe manifestation of *Mycobacterium tuberculosis *infection and is associated with significant morbidity and mortality. Its clinical and radiologic presentations are often nonspecific and may mimic malignancy, inflammatory disorders, or fungal infections, frequently resulting in diagnostic delay. Conventional cerebrospinal fluid (CSF) testing has limited sensitivity, and definitive diagnosis may require histopathologic confirmation when noninvasive studies are inconclusive. We report the case of a 76-year-old woman with a history of cerebrovascular accident and hypertension who presented with a six-month history of progressive encephalopathy following travel to Ghana. Initial neuroimaging was unrevealing, while CSF analysis demonstrated lymphocytic pleocytosis with markedly low glucose; routine meningitis testing was negative. Subsequent brain magnetic resonance imaging (MRI) revealed diffuse nodular leptomeningeal enhancement involving the posterior fossa and brainstem. Given high clinical suspicion, empiric antituberculous therapy was initiated. Definitive diagnosis was achieved through open meningeal biopsy, which demonstrated necrotizing granulomas with acid-fast bacilli, confirming CNS TB. No evidence of pulmonary TB was identified. The patient demonstrated significant clinical and radiologic improvement with continued antituberculous therapy and adjunctive corticosteroids. This case highlights the diagnostic complexity of CNS TB and emphasizes the importance of histopathologic confirmation when noninvasive testing is nondiagnostic. Early recognition of CNS TB in patients with chronic meningitis, atypical neuroimaging findings, and relevant epidemiologic exposure is critical for timely treatment and improved neurologic outcomes.

## Introduction

Central nervous system (CNS) tuberculosis (TB) is a rare but serious form of extrapulmonary TB, accounting for approximately 1% of all TB cases and 5-10% of extrapulmonary TB cases worldwide [1-3]. Despite its relative rarity, CNS TB contributes disproportionately to neurologic morbidity and mortality because of delayed diagnosis and the potential for irreversible neurologic injury [1-3]. The disease spectrum includes tuberculous meningitis, intracranial tuberculomas, and isolated leptomeningeal involvement [3]. Clinical manifestations are often insidious and nonspecific, ranging from headache and cognitive decline to progressive encephalopathy, frequently leading to delayed diagnosis [4].

Although CNS TB is classically associated with constitutional symptoms or evidence of systemic TB, isolated CNS involvement without pulmonary disease may occur and can present significant diagnostic challenges. In older adults, progressive cognitive decline or encephalopathy may be the predominant manifestation, mimicking neurodegenerative, inflammatory, or neoplastic disorders and delaying recognition of an underlying infectious etiology.

Diagnostic evaluation of CNS TB remains challenging. Cerebrospinal fluid (CSF) cultures and nucleic acid amplification tests demonstrate limited sensitivity due to the paucibacillary nature of the disease [5]. Neuroimaging findings, although suggestive, are not pathognomonic and may overlap with other disease processes. Leptomeningeal enhancement and nodular inflammatory changes may be seen in neurosarcoidosis, leptomeningeal carcinomatosis, fungal infections such as central nervous system histoplasmosis, or other granulomatous conditions [6,7]. Given this diagnostic uncertainty, empiric antituberculous therapy is often initiated based on clinical suspicion and epidemiologic risk factors [8]. Additionally, intracranial tuberculomas and leptomeningeal involvement have been reported in elderly patients and may further complicate radiologic differentiation from neoplastic and inflammatory conditions [9].

When noninvasive testing and empiric treatment fail to establish a definitive diagnosis, histopathologic confirmation through brain or meningeal biopsy remains the gold standard despite its invasive nature [10]. We present a case of biopsy-proven isolated CNS TB in an elderly woman who presented with progressive encephalopathy without pulmonary involvement or other systemic manifestations of TB, highlighting the diagnostic challenges associated with this uncommon presentation.

## Case presentation

A 76-year-old woman with a medical history significant for cerebrovascular accident, hypertension, and prior lower-extremity deep vein thrombosis on chronic anticoagulation presented with acute agitation and altered mental status. Family members reported a gradual cognitive decline over the preceding six months following travel to Ghana. She had no history of tobacco or alcohol use. The patient denied respiratory symptoms, including cough, hemoptysis, and dyspnea, as well as constitutional symptoms such as fever, night sweats, or unintentional weight loss. 

The patient's primary epidemiologic risk factor was frequent travel to Ghana, which she visited approximately twice yearly while staying with family. HIV testing was nonreactive, and QuantiFERON-TB Gold testing (QIAGEN N.V., Venlo, Netherlands) was indeterminate. Evaluation for systemic TB was unrevealing. Admission chest radiography demonstrated no evidence of active pulmonary disease or findings suggestive of pulmonary TB. No known TB exposures were identified other than travel to a TB-endemic region. Family members and close contacts were not reported to have symptoms suggestive of active TB.

The patient underwent a comprehensive evaluation for encephalopathy. Initial laboratory studies included a complete blood count, inflammatory markers, renal function testing, urinalysis, urine culture, and urine toxicology screening (Table 1). These studies were unrevealing and did not identify an alternative etiology for her encephalopathy.

**Table 1:** Initial laboratory evaluation Results were largely unremarkable and did not identify an alternative metabolic, infectious, inflammatory, or toxicologic cause of the patient's presentation. ESR, erythrocyte sedimentation rate; CRP, C-reactive protein; BUN, blood urea nitrogen; eGFR, estimated glomerular filtration rate; HPF, high-power field; THC, tetrahydrocannabinol.

Test	Result	Reference Range
Complete Blood Count		
White Blood Cell Count	6.0 ×10³/μL	4.0–11.0 ×10³/μL
Hemoglobin	12.0 g/dL	12.0–16.0 g/dL
Platelet Count	250 ×10³/μL	150–450 ×10³/μL
Inflammatory Markers		
ESR	2 mm/hr	<30 mm/hr
CRP	0.1 mg/dL	<0.5 mg/dL
Renal Function		
BUN	10 mg/dL	7–20 mg/dL
Creatinine	1.0 mg/dL	0.6–1.3 mg/dL
eGFR	70 mL/min/1.73 m²	>60 mL/min/1.73 m²
Urinalysis		
Appearance	Clear	Clear
Color	Colorless	Yellow–Amber
pH	7.0	5.0–8.0
Specific Gravity	1.008	1.010–1.030
Blood	Negative	Negative
Leukocyte Esterase	Negative	Negative
Nitrite	Negative	Negative
Protein	Negative	Negative
Glucose	Normal	Negative
Ketones	Negative	Negative
Bilirubin	Negative	Negative
Urobilinogen	Normal	Normal
Red Blood Cells	<1 /HPF	0–2 /HPF
White Blood Cells	1 /HPF	0–5 /HPF
Microbiology		
Urine Culture	No Growth	No Growth
Urine Toxicology Screen		
Amphetamines	Negative	Negative
Benzodiazepines	Negative	Negative
Fentanyl	Negative	Negative
Cannabinoids (THC)	Negative	Negative

Neurologic examination demonstrated generalized weakness without focal motor or sensory deficits. Mental status assessment was notable for encephalopathy and agitation requiring close neurologic monitoring. The patient was admitted to a step-down unit and underwent frequent neurologic assessments throughout her hospitalization. No meningeal signs were documented. She was evaluated by speech-language pathology and was cleared for a regular diet without evidence of significant swallowing dysfunction.

Initial non-contrast computed tomography (CT) of the head was unremarkable, and CT angiography demonstrated no large-vessel occlusion. Magnetic resonance imaging (MRI) of the brain with contrast demonstrated diffuse nodular leptomeningeal enhancement predominantly involving the posterior fossa and brainstem, with additional supratentorial involvement (Figure 1). Given concern for possible neuraxial involvement, MRI of the cervical, thoracic, and lumbar spine was also obtained and was unremarkable, demonstrating no evidence of spinal cord compression, nerve root involvement, or findings suggestive of spinal tuberculous arachnoiditis.

**Figure 1:**
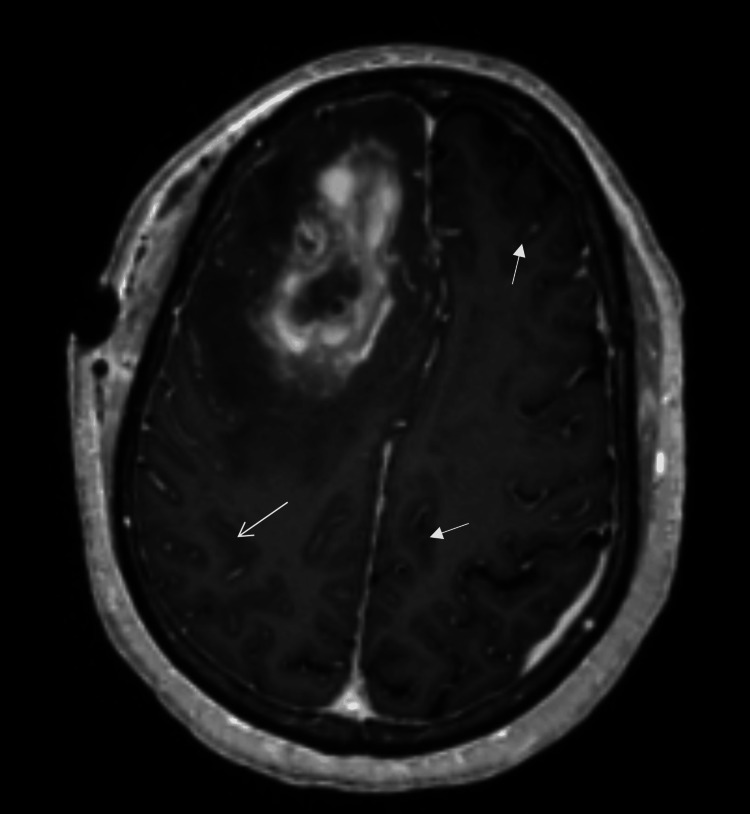
Axial contrast-enhanced T1-weighted brain MRI demonstrating diffuse supratentorial leptomeningeal enhancement. The plain arrow indicates diffuse leptomeningeal enhancement along the cerebral convexities, while arrows with white arrowheads indicate additional nodular supratentorial leptomeningeal enhancement.

Lumbar puncture revealed findings consistent with chronic meningitis, including lymphocytic pleocytosis, elevated protein concentration, and markedly reduced glucose levels. Serial cerebrospinal fluid analyses are summarized in Table 2. Additional CSF studies, including Gram stain, Venereal Disease Research Laboratory (VDRL) test, cryptococcal antigen testing, blastomycosis studies, meningitis/encephalitis polymerase chain reaction (PCR) testing, and a comprehensive autoimmune/paraneoplastic encephalitis evaluation, were negative.

**Table 2:** Cerebrospinal fluid (CSF) analysis and diagnostic studies Serial CSF analyses demonstrated persistent lymphocytic pleocytosis and marked hypoglycorrhachia consistent with chronic granulomatous meningitis. Extensive infectious, autoimmune, and paraneoplastic investigations were unrevealing. Despite characteristic CSF abnormalities suggestive of tuberculous meningitis, definitive diagnosis ultimately required histopathologic confirmation through meningeal biopsy. CSF, cerebrospinal fluid; WBC, white blood cell count; RBC, red blood cell count; VDRL, Venereal Disease Research Laboratory test; PCR, polymerase chain reaction.

Parameter	Initial Hospitalization	Prior to Discharge	Reference Range
Tube Number	2	3	—
Volume (mL)	13.0	7.0	—
Color	Colorless	Colorless	Colorless
Appearance	Slightly Cloudy	Slightly Cloudy	Clear
WBC (/μL)	230	164	0–5
RBC (/μL)	13	99	0–5
Neutrophils (%)	2	6	0–6
Lymphocytes (%)	96	80	40–80
Monocytes (%)	2	9	15–45
Eosinophils (%)	0	5	0–1
Glucose (mg/dL)	21	20	40–70
Gram Stain	No WBCs seen; No organisms seen	No WBCs seen; No organisms seen	Negative
CSF VDRL	Negative	Negative	Negative
Meningitis/Encephalitis PCR Panel	Negative	Negative	Negative
CSF Cryptococcal Antigen	Negative	Negative	Negative
CSF Blastomycosis Studies	Negative	Negative	Negative
Autoimmune/Paraneoplastic Encephalitis Panel*	Negative	Negative	Negative

Serial CSF analyses demonstrated persistent lymphocytic pleocytosis with a predominantly lymphocytic differential and marked hypoglycorrhachia. Despite extensive infectious, autoimmune, and paraneoplastic investigations, no alternative diagnosis was identified.

Given the patient's epidemiologic exposure, characteristic neuroimaging findings, and CSF profile suggestive of chronic tuberculous meningitis, empiric antituberculous therapy with rifampin, isoniazid, pyrazinamide, and ethambutol (RIPE) was initiated. Adjunctive corticosteroid therapy was also started because of concern for CNS TB with meningeal involvement and the potential for inflammatory-mediated neurologic injury.

Interval MRI demonstrated persistent leptomeningeal enhancement throughout the posterior fossa (Figure 2). Given ongoing diagnostic uncertainty, neurosurgical consultation was obtained, and an open meningeal biopsy was performed. Histopathologic examination of the meningeal biopsy demonstrated necrotizing granulomatous inflammation with positive acid-fast bacilli (AFB) staining. Additional immunohistochemical and special stains did not support malignancy or alternative infectious etiologies. No evidence of malignancy was identified. Collectively, these findings established the diagnosis of CNS TB.

**Figure 2:**
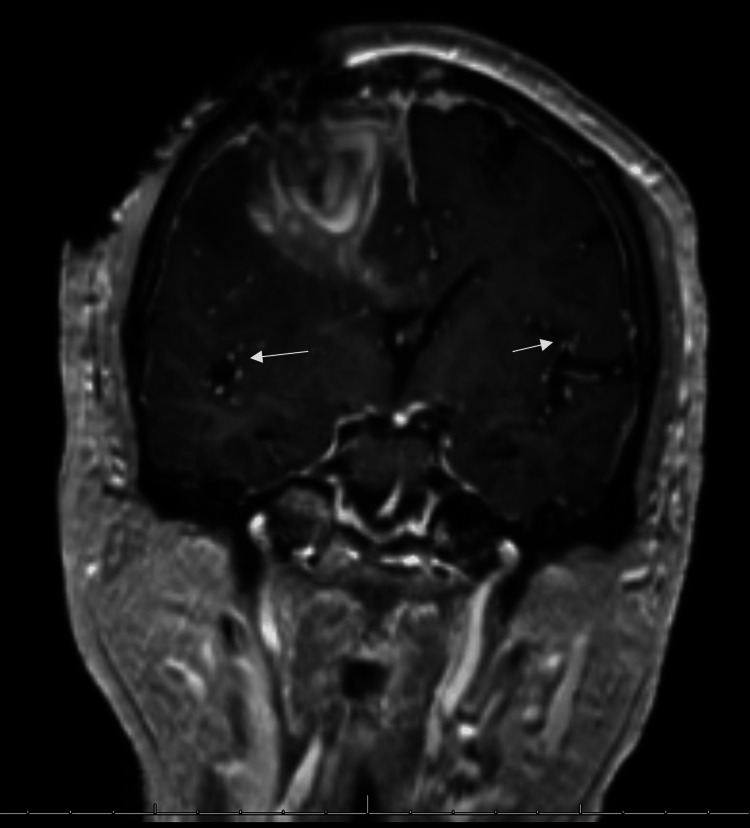
Axial contrast-enhanced T1-weighted brain MRI demonstrating persistent posterior fossa leptomeningeal enhancement throughout the posterior fossa on interval imaging (arrows), consistent with an ongoing infectious or inflammatory process prior to histopathologic diagnosis.

The patient underwent a prolonged hospitalization for diagnostic evaluation and management. Following initiation of RIPE therapy and adjunctive corticosteroids, her clinical status gradually improved, and she was ultimately discharged with plans for continued outpatient antituberculous treatment and follow-up. MRI at the six-month follow-up showed interval improvement of the previously identified leptomeningeal abnormalities (Figure 3).

**Figure 3:**
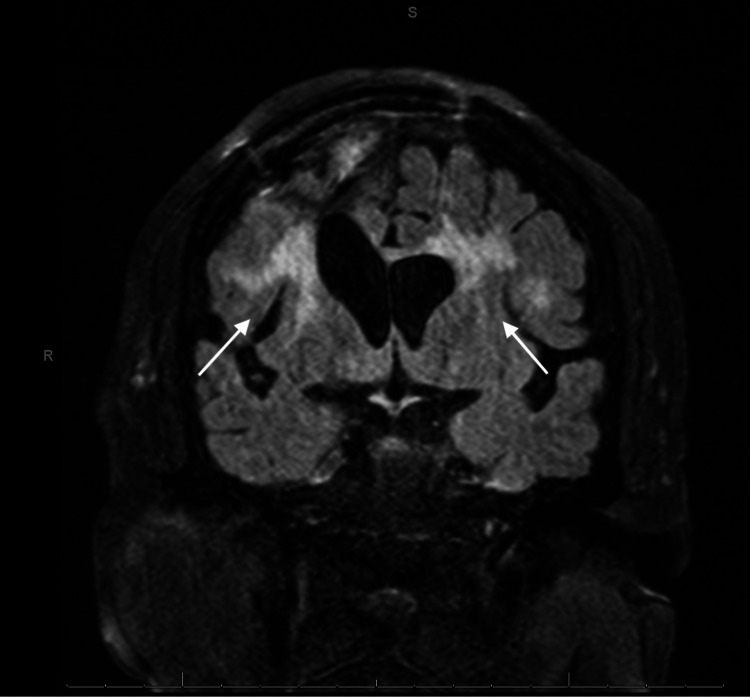
FLAIR MRI (coronal view) at the six-month follow-up Arrows indicate the regions corresponding to the prior leptomeningeal enhancement seen on earlier contrast-enhanced MRI studies, demonstrating marked interval improvement following treatment. FLAIR, fluid-attenuated inversion recovery

## Discussion

This case highlights the diagnostic challenges associated with isolated CNS TB, particularly in elderly patients presenting with progressive encephalopathy in the absence of pulmonary involvement or other systemic manifestations of TB. In older adults, progressive cognitive decline and encephalopathy are often attributed to metabolic, vascular, neurodegenerative, or neoplastic processes. Consequently, infectious etiologies such as CNS TB may not be initially considered, particularly when constitutional symptoms and pulmonary manifestations are absent. The absence of fever, weight loss, night sweats, or respiratory symptoms in this patient broadened the differential diagnosis and contributed to the complexity of the diagnostic evaluation.

Neuroimaging played a critical role in raising suspicion for CNS TB. The diffuse nodular leptomeningeal enhancement with posterior fossa and brainstem predominance observed on contrast-enhanced MRI reflects basal meningeal involvement, a recognized feature of CNS TB [7]. However, leptomeningeal enhancement is a nonspecific imaging finding with a broad differential diagnosis. Infectious etiologies include TB, fungal meningitis, and chronic bacterial infections, whereas noninfectious causes include neurosarcoidosis, autoimmune inflammatory disorders, and leptomeningeal carcinomatosis from metastatic malignancy [6,7]. Persistent posterior fossa leptomeningeal enhancement on interval imaging further supported an infectious or inflammatory process while illustrating the substantial radiographic overlap among these entities [7,9].

Consistent with prior reports, microbiologic confirmation through CSF analysis proved limited because of the paucibacillary nature of CNS TB [1,3]. Although the patient's CSF profile was highly suggestive of chronic tuberculous meningitis, extensive infectious, autoimmune, and paraneoplastic investigations failed to establish a definitive diagnosis. In this setting, histopathologic confirmation through open meningeal biopsy was essential both for establishing the diagnosis and for excluding alternative etiologies such as neurosarcoidosis and leptomeningeal carcinomatosis [10].

The educational significance of this case lies not in the CSF or imaging findings themselves, which are well-described manifestations of CNS TB, but in the presentation of biopsy-proven isolated CNS TB as progressive encephalopathy in the absence of pulmonary disease, constitutional symptoms, or known TB exposure. The extensive differential diagnosis, nondiagnostic noninvasive testing, and ultimate requirement for histopathologic confirmation illustrate the challenges clinicians may encounter when evaluating chronic meningitis in older adults. This case further underscores the importance of maintaining a high index of suspicion for CNS TB in patients with progressive neurologic decline and compatible epidemiologic risk factors, even when routine diagnostic studies are unrevealing.

As described in the case presentation, the patient's clinical recovery, functional improvement during rehabilitation, and interval radiographic improvement on six-month follow-up MRI following treatment with RIPE, together with adjunctive corticosteroid therapy, further support the diagnosis and are consistent with established management recommendations for CNS TB [8].

## Conclusions

This case highlights the diagnostic challenges of isolated CNS TB presenting as progressive encephalopathy in the absence of pulmonary disease, constitutional symptoms, or known TB contacts. Although neuroimaging and CSF findings raised suspicion for chronic granulomatous meningitis, these findings were insufficient to establish a definitive diagnosis and overlapped with a broad differential that included infectious, inflammatory, autoimmune, and neoplastic disorders. Extensive laboratory, microbiologic, radiographic, and cerebrospinal fluid evaluation failed to establish a definitive diagnosis, ultimately necessitating histopathologic confirmation through meningeal biopsy.

This report underscores the importance of maintaining a high index of suspicion for CNS TB in patients with chronic meningitis, progressive cognitive decline, and relevant epidemiologic risk factors, even when routine microbiologic studies are unrevealing and pulmonary involvement is absent. Early recognition and prompt initiation of antituberculous therapy are essential to prevent irreversible neurologic injury. The patient's favorable clinical recovery, functional improvement during rehabilitation, and radiographic response to therapy further illustrate the potential benefits of timely diagnosis and treatment.
